# Editorial: Public health, suicide, and substance addiction

**DOI:** 10.3389/fpubh.2023.1343708

**Published:** 2023-12-19

**Authors:** Sheikh Mohd Saleem, Muhammad Alkasaby, Sheikh Shoib

**Affiliations:** ^1^UNICEF, New Delhi, India; ^2^Centre for Global Mental Health, London School of Hygiene and Tropical Medicine, University of London, London, United Kingdom; ^3^Directorate of Health Services Kashmir, Srinagar, Jammu and Kashmir, India

**Keywords:** suicide prevention, substance addiction, public mental health (PMH), addiction, mental health

## Background

Suicide remains a critical public health concern, intricately entwined with factors ranging from mental health vulnerabilities to societal pressures. Research on “*Public health, suicide, and substance addiction*” offers a wealth of information about the complex interplay between these three problems. Suicide is a hidden epidemic that is quiet and has many underlying factors. Scholars have traditionally placed a high value on investigating and evaluating various causative factors (1).

In this editorial, we navigate through the key themes and notable findings across a diverse array of studies, each shedding light on distinct facets of this complex landscape.

## Understanding self-harm and suicide

The exploration of deliberate self-harm and suicide emerges as a central theme in our collection of research. The study by Devassy et al. unearths the unexplored territory of bio-psychosocial vulnerabilities and stressors leading to self-harm, particularly within the Indian context. Their findings underscore the dormant nature of biopsychosocial vulnerabilities until activated by life stressors, resulting in severe self-harm behaviors. The implications suggest that mental health team-driven assertive engagement and positive coping interventions are vital in preventing reattempts.

Likewise, Chen et al. delve into the relationship between academic stress, school bullying, and self-harm behaviors among Chinese middle school students. This study not only identifies common factors but also unravels the mediating role of anxiety and depression in the association of school bullying and academic stress with self-harm. The results advocate for strategies to reduce academic pressure, prevent school bullying, and evaluate and intervene in negative emotions among middle school students.

## Risk factors and interventions

In the realm of substance addiction, Tang et al. present a risk assessment for relapse among participants in methadone maintenance treatment. The study employs an innovative approach, utilizing group-LASSO-based Bayesian networks to identify risk factors and assess relapse risk based on treatment duration. The implications stress the necessity of targeted interventions and education tailored to the treatment duration of individuals, thereby mitigating the relapse rate.

Yu et al. investigate influencing factors of suicidal ideation in lung cancer patients. The study not only reveals a higher incidence of suicidal ideation among lung cancer patients but also highlights the need for routine screening and assessment. The findings suggest that addressing suicidal ideation requires a comprehensive understanding of physiological, psychological, and social factors.

## Predictive models for suicidal ideation

Predictive models for suicidal ideation take center stage in two studies. Liao et al. developed a radial basis function neural network for predicting suicidal ideation among Chinese college students, achieving high accuracy. Meanwhile, Zheng et al. identify risk factors for self-poisoning suicide and present a nomogram for predicting self-poisoning suicide among poisoned patients. Both studies emphasize the accuracy and potential utility of predictive models in identifying high-risk individuals, and facilitating early intervention and prevention strategies.

## Mental health services and suicide risk

Lee et al. explore the suicide risk of psychiatric patients following recent health care service utilization in South Korea. The study underscores the significance of suicide prevention within clinical settings. The findings call for heightened awareness and preventive measures, particularly considering the increased suicide risk associated with recent psychiatric and non-psychiatric admission and outpatient visits.

## Industry-specific challenges

The unique challenges faced by individuals in the Australian Construction Industry are eloquently detailed by Tyler et al.. The study sheds light on the drivers and experiences of suicidal ideation among industry workers, emphasizing the necessity for industry-specific prevention strategies. The identified themes, ranging from work-related challenges to personal issues, underscore the importance of tailored interventions and support programs within specific occupational contexts.

## Suicide literacy and stigma

Jahan et al. examine suicide literacy and stigma among young adults in Bangladesh. The study highlights the need for awareness programs to enhance knowledge and decrease the stigma surrounding suicide. The findings suggest that addressing suicide literacy is integral to fostering a more supportive societal environment, enabling early intervention and destigmatization.

## Suicidal ideation in cancer patients

The prevalence of suicidal ideation and attempts among people with cancer is explored by Nigussie et al. in Eastern Ethiopia. The study unveils a concerning magnitude of suicidal ideation and attempts among cancer patients, emphasizing the need for early screening, diagnosis, and treatment of suicide risk factors. The identified risk factors, including living alone, depressive symptoms, and poor social support, underscore the importance of a holistic approach to mental health care for cancer patients.

## Conclusion

This collection of research articles provides a comprehensive panorama of the intricate dynamics surrounding public health, suicide, and substance addiction. The themes explored—from the understanding of self-harm and suicide to predictive models, risk factors, and industry-specific challenges—collectively contribute to our evolving comprehension of these complex issues. The implications for preventive strategies, tailored interventions, and heightened awareness underscore the pivotal role of public health initiatives in addressing the intricate interplay of factors contributing to suicide and substance addiction. As we navigate these challenges, the insights gleaned from these studies pave the way for a more informed, compassionate, and effective approach to public health in the context of suicide and substance addiction.

## Author contributions

SMS: Conceptualization, Investigation, Methodology, Project administration, Supervision, Validation, Visualization, Writing—original draft, Writing—review & editing. MA: Investigation, Methodology, Visualization, Writing—review & editing. SS: Data curation, Investigation, Methodology, Resources, Validation, Visualization, Writing—review & editing.
